# Estimating Multivariate Discrete Distributions Using Bernstein Copulas †

**DOI:** 10.3390/e20030194

**Published:** 2018-03-14

**Authors:** Victor Fossaluza, Luís Gustavo Esteves, Carlos Alberto de Bragança Pereira

**Affiliations:** Institute of Mathematics and Statistics, Universidade de São Paulo, São Paulo, SP 05508-090, Brazil

**Keywords:** Bernstein polynomial, copula, nonparametric inference, Aitchison’s distance

## Abstract

Measuring the dependence between random variables is one of the most fundamental problems in statistics, and therefore, determining the joint distribution of the relevant variables is crucial. Copulas have recently become an important tool for properly inferring the joint distribution of the variables of interest. Although many studies have addressed the case of continuous variables, few studies have focused on treating discrete variables. This paper presents a nonparametric approach to the estimation of joint discrete distributions with bounded support using copulas and Bernstein polynomials. We present an application in real obsessive-compulsive disorder data.

## 1. Introduction

The association between random variables is a subject of interest in many scientific fields. The most complete method of characterizing the association between random variables is to determine the joint distribution of these random variables. Multivariate density functions, for absolutely continuous variables, and multivariate probability mass functions, for discrete variables, have become the focus of researchers interested in evaluating such associations (see, for example, [1,2,3,4]).

The motivation for the present paper was a study performed as part of the Obsessive-Compulsive Spectrum Disorder Program of the Institute of Psychiatry, University of São Paulo Medical School. A group of 1001 consecutive adult outpatients diagnosed with primary obsessive-compulsive disorder (OCD) according to the DSM-IV criteria [5] were recruited, and some of these patients were submitted to psychiatric treatment. Their OCD severity was evaluated using the Yale-Brown Scale (YBOCS; [6,7]) at the beginning of the project. At the time when the data records were accessed, only 213 patients participated in the re-evaluation using the same scale. The YBOCS is composed of two sub-scales, obsession (O) and compulsion (C), and each sub-scale assumes values in the set of integers {0,1,…,20}. To measure the OCD severity of the patients, we considered the maximum value between the O and C sub-scale measures, max{O;C}; this method of scoring is known as the M-YBOCS scale (see the discussions in [8,9]).

**Figure 1:**
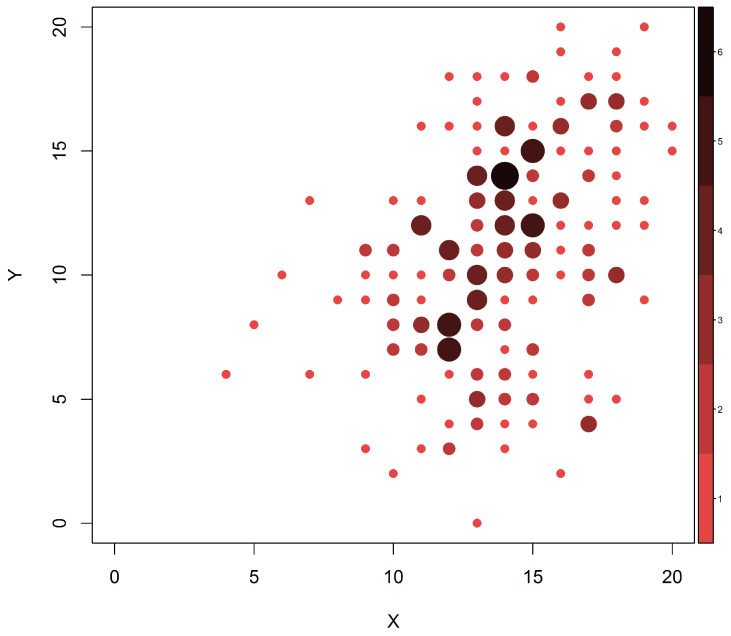
Frequencies of each observed OCD severity before and after treatment.

Figure 1 presents the initial (*X*) and final (*Y*) M-YBOCS scores for all 213 patients for whom both initial and final scores were obtained. In this graphic, darker colors and larger dots represent higher cell frequencies. Our first objective is to estimate the marginal distributions of the initial and final scores. For this purpose, all available information should be used: all 1001 patients included in the first evaluation and all 213 remaining patients at the end of the study. If we use only the complete pairs of observation, omitting missing marginal values, we obtain only 213 pairs of measurements to be used in the estimation of the joint distribution of interest, the support of which possibly contains $441(=21^{2})$ points, nearly double the sample size. As a consequence, standard methods of estimation (like maximum likelihood) would unavoidably yield estimates equal to zero for most cell probabilities. It is then reasonable to consider the whole dataset (including the available incomplete pairs) in order to improve such estimates.

The objective of the present paper is to introduce a method of estimating multivariate discrete probability mass functions in the presence of (marginal) missing data. For this purpose, we developed an estimation method that uses both empirical distribution functions and Bernstein polynomials. The procedure consists of estimating a smooth joint distribution function, followed by applying a method that transforms this function into a discrete function, i.e., the estimated joint probability mass function. The results of this new method are compared with those of alternative methods, both graphically and by evaluating standard distances.

Section 2 describes the existing methods found in the literature that will be considered for comparison. Section 3 describes our estimator for the joint probability functions. Section 4 presents a discussion of the new method and comparisons of this method to the alternative methods using both simulated samples and the real OCD example. Finally, in Section 5, we present our final comments and considerations for future work.

## 2. Existing Solutions

First, we introduce the mathematical framework for our problem. Let *F* be the unknown distribution function of a random vector X that takes values in a subset of $R^{p}$. A sample of size *n* of X is represented by $X_{1},\ldots ,X_{n}$, where $X_{i}=\left(X_{i1},\ldots ,X_{ip}\right)$ and i=1,…,n. In other words, the $X_{i}$’s are conditionally independent and identically distributed random variables, given any distribution function *F*. Observations of $X_{i}$ are denoted by $x_{i}$.

Assuming that the distribution *F* is drawn from a known family of distributions, we represent the statistical model by (X,F,P), where X is the sample space, F is a sigma-algebra of its subsets and $P=\left\{P(\cdot |\theta ):\theta \in \Theta \right\}$ is a family of distributions indexed by the parameter θ that belongs to the parameter space Θ. The estimation of *F* is then reduced to that of the parameter θ, and the dependence structure is limited to that supported by the underlying statistical model. For many years, the multivariate normal distribution has been used for most multivariate analyses (see, for example, [10,11]). Recently, for many random phenomena whose distributions are skewed and possess heavier tails than those of the normal distribution, alternative distributions, such as multivariate skew-elliptical distributions, have been adopted [12,13].

In recent approaches, copulas have become a popular tool for modeling multivariate dependence structures and for obtaining new multivariate distributions with given marginals. In short, a copula is a multivariate distribution whose marginals are uniform over the entire range [0,1]. There are many parametric families of copulas, allowing for the modeling of many different dependence structures [1,2,3,4]. Let *F* be a *p*-dimensional distribution function with the margins $F_{1},\ldots ,F_{p}$. Sklar [14] first showed that there exists a *p*-dimensional copula *C* such that:

$$F\left(x_{1},\ldots ,x_{p}\right)=C\left(F_{1}\left(x_{1}\right),\ldots ,F_{p}\left(x_{p}\right)\right)$$

for all $x=(x_{1},\ldots ,x_{p})$ in the domain of *F*. If the variables $X_{1},\ldots ,X_{p}$ are absolutely continuous, then the copula *C* is unique; otherwise, *C* is uniquely determined on $Ran\left(F_{1}\right)\times \ldots \times Ran\left(F_{p}\right)$, where $Ran(F_{i})$ is the image of the function $F_{i}$, i=1,…,p [14]. Thus, the copula can be used to separately model the margins and the dependence structure. The non-unique representation of a copula for discrete distributions is a theoretical issue that must be considered in the context of an analytical proof, but this does not limit its empirical applications [15]. However, the above theorem [14] does not tell us how to find the copula *C*. This problem is widely discussed in the literature, and several solutions to this problem have been proposed (see, for example, [16]). The most widely-used approach is to adjust several families of (parametric) copulas and choose one of them using certain selection criteria or a goodness-of-fit test [17,18,19,20,21].

Nonparametric techniques may also be applied to estimate a multivariate distribution. A popular solution using this approach is the application of the empirical distribution function $F^{\left(n\right)}:R^{p}\to \left[0,1\right]$, which is defined, for $\left(t_{1},\ldots ,t_{p}\right)\in R^{p}$, as:

(1) $$F^{\left(n\right)}\left(t_{1},\ldots ,t_{p}\right)=\frac{1}{n}\sum_{i=1}^{n}I\left\{x_{1i}\leq t_{1},\ldots ,x_{pi}\leq t_{p}\right\},$$

where I{A} is the indicator of the set *A*. This approach is equivalent to using the relative frequencies to estimate the joint probability mass function. The relative frequencies coincide with the maximum-likelihood estimate under the assumption that the data are drawn from a multinomial distribution. One shortcoming of such approaches is that the probability of any non-observed cells will be estimated to be zero.

Another possible approach is to use some function to smooth the empirical distribution. We can consider the Bernstein polynomials [22,23] for this purpose because of their simplicity and good mathematical properties [24,25]. Let $h:{\left[0,1\right]}^{p}\to R$ be a continuous function. The *m*^th^-degree (multivariate) Bernstein polynomial for the function *h*, namely, $B_{h}^{m}:{\left[0,1\right]}^{p}\to R$, is defined as:

(2) Bhm(x1,…,xp)=∑j1=0m…∑jp=0mhj1m,…,jpm∏i=1pmjixiji(1−xi)m−ji.

The multivariate Bernstein polynomials for the function *h* converge uniformly to the function *h* as m→∞ [26,27], and its derivatives are simple to obtain. The function *h* must be defined in ${\left[0,1\right]}^{p}$, and therefore, for practical purposes, data that do not take values in ${\left[0,1\right]}^{p}$ must first be transformed [24]. To apply this method to the OCD data, for example, we consider the transformation Y=X/20. Moreover, the polynomial degree adopted here is m=n/log(n), as suggested by [24]. Bernstein polynomials have been used to approximate a copula *C* by simply replacing the function *h* with the copula. The resulting Bernstein polynomial, $B_{C}^{m}$, which is also a copula that strongly converges to *C*, is called a Bernstein copula [28,29,30,31,32]. When the true copula is unknown, the empirical copula can be used instead, and the resulting function is called the empirical Bernstein copula [16,33,34,35,36,37]. The empirical copula is defined as:

$$C_{n}\left(u_{1},\ldots ,u_{p}\right)=\frac{1}{n}\sum_{i=1}^{n}I\left\{F_{1}\left(x_{1i}\right)\leq u_{1},\ldots ,F_{p}\left(x_{pi}\right)\leq u_{p}\right\}.$$

Note that even when $F_{i}$, i∈1,…,n, is unknown, we can use the empirical marginal distribution $F_{i}^{\left(n\right)}$ as a consistent estimator of $F_{i}$, according to the Glivenko–Cantelli theorem (e.g., [38]). Other estimators for marginal distributions could be considered instead, as in the procedure proposed in the next section.

We have so far obtained a continuous function as an estimate while our objective is clearly to estimate a (discrete) probability mass function. Hence, this function must be discretized to obtain an adequate estimate. This can be achieved as follows: suppose, with no loss of generality, that $X=\left(X_{1},\ldots ,X_{p}\right)$ is a random vector such that all its components $X_{i}$, i=1,2,…,p, assume values in the set Ω={0,1,…,k} with probability one. In addition, there always exists a continuous random vector $Z=(Z_{1},\ldots ,Z_{p})$ with distribution function *F* such that $P(0\leq Z_{i}\leq k)=1$, i=1,…,p, and $X_{i}=\sum_{j=0}^{k}j\;I\left\{j-0.5<Z_{i}\leq j+0.5\right\}$, *i*. It follows that:

$$P\left(X_{1}=x_{1},\ldots ,X_{p}=x_{p}\right)=P\left(x_{1}-0.5<Z_{1}\leq x_{1}+0.5,\ldots ,x_{p}-0.5<Z_{p}\leq x_{p}+0.5\right).$$

Let *F* (or an estimate $\hat{F}$) be the continuous joint distribution function of the random vector Z, and let $B=\left[a,b\right]=\left[a_{1},b_{1}\right]\times \ldots \times \left[a_{p},b_{p}\right]$ be a *p*-dimensional rectangle with all its vertices in Ω. The *F*-volume of *B* [4] is then given by:

(3) $$V_{F}\left(B\right)=\sum_{c}sgn\left(c\right)F\left(c\right),$$

where the sum is taken over all vertices $c=(c_{1},\ldots ,c_{p})$ of *B*, and sgn(c) is given by:

$$sgn\left(c\right)=\left\{\begin{array}{cc} 1, & \mathrm{if}\;c_{j}=a_{j}\;\mathrm{for}\;\mathrm{an}\;\mathrm{even}\;\mathrm{number}\;\mathrm{of}\;j^{\prime }s,\\ -1, & \mathrm{if}\;c_{j}=a_{j}\;\mathrm{for}\;\mathrm{an}\;\mathrm{odd}\;\mathrm{number}\;\mathrm{of}\;j^{\prime }s. \end{array}\right.$$

In particular, suppose $b=\left(b_{1},\ldots ,b_{p}\right)\in {\left\{0,1,\ldots ,k\right\}}^{p}$, and take $B=\left[b-\frac{1}{2}\;1,b+\frac{1}{2}\;1\right]=\left[b_{1}-0.5,b_{1}+0.5\right]\times \left[b_{2}-0.5,b_{2}+0.5\right]\times \ldots \times \left[b_{p}-0.5,b_{p}+0.5\right]$, with $b_{i}\in \Omega$, ∀i=1,…,p, then the probability of the event $\left\{X=b\right\}=\left\{X_{1}=b_{1},\ldots ,X_{p}=b_{p}\right\}$ can be calculated (estimated) as:

$$P\left(X=b\right)=P\left(b_{1}-0.5<Z_{1}\leq b_{1}+0.5,\ldots ,b_{p}-0.5<Z_{p}\leq b_{p}+0.5\right])=V_{F}\left(B\right).$$

Because weak convergence occurs at the points of continuity of the limiting distribution function *F* and because our goal is to estimate a discrete probability mass function, we consider sets of the form $B=[b-\frac{1}{2}\;1,b+\frac{1}{2}\;1]$, with $b_{i}\in \Omega$, so that the vertices of the *p*-dimensional rectangle *B* are always points of continuity of the distribution function of the discrete random vector X. Thus, such discretization yields satisfactory estimates for the probability mass function of X.

## 3. Proposed Solutions

Our proposed method for estimating the joint distribution of a discrete random vector consists of using Bernstein polynomials to estimate both the marginals and the copula. The advantage of this method is that it allows all observations to be used, even in the case of missing values in some variable. Furthermore, this method is a nonparametric approach, and there are few restrictions on the dependence structure.

First, for each random variable $X_{i}$, we estimate the marginal distributions using the empirical marginal distribution with $n_{i}$ observations, $F_{i}^{\left(n_{i}\right)}\left(x\right)=\frac{1}{n_{i}}\sum_{j=1}^{n_{i}}I\left(x_{ij}\leq x\right)$, i=1,…,p; then, the Bernstein polynomial of degree $m_{i}=n_{i}/log\left(n_{i}\right)$ is used to smooth this function:

Bimi(x)=∑j=1miFi(ni)jmimijxj(1−x)(mi−j).

As this estimator converges to the marginal distribution [24], we estimate the copula using an alternative version of the empirical copula based on the *n* complete observations and the estimates $B_{i}^{m_{i}}$, i=1,…,p,

$$C_{n}\left(u_{1},\ldots ,u_{p}\right)=\frac{1}{n}\sum_{j=1}^{n}I\left\{B_{1}^{m_{1}}\left(x_{1j}\right)\leq u_{1},\ldots ,B_{p}^{m_{p}}\left(x_{pj}\right)\leq u_{p}\right\},$$

and smooth this function to obtain the corresponding empirical Bernstein copula,

BCnm(u1,…,up)=∑j1=0m…∑jp=0mCnj1m,…,jpm∏i=1pmjixiji(1−xi)m−ji.

Note that the construction of the copula $C_{n}$ using Bernstein polynomials rather than empirical (marginal) distribution functions yields, at least in the examples to be presented in Section 4, non-zero estimates for non-observed cells. This feature justifies the choice of this alternative version of the empirical copula.

The estimate of the joint distribution function is a discretization (Equation (3)) of the following function:

(4) $${\hat{F}}_{m,n}\left(x_{1},\ldots ,x_{p}\right)=B_{C_{n}}^{m}\left(B_{1}^{m}\left(x_{1}\right),\ldots ,B_{p}^{m}\left(x_{p}\right)\right).$$

The algorithm used to obtain the proposed solution is quite simple and is summarized below:

1. for all $n_{i}$ observations of each variable $X_{i}$, estimate the marginal empirical distribution function $F_{i}^{\left(n_{i}\right)}$;
2. smooth each function $F_{i}^{\left(n_{i}\right)}$ using a Bernstein polynomial $B_{i}^{\left(m_{i}\right)}$ of degree $m_{i}$;
3. for all complete observations of the random vector X, estimate the empirical copula $C_{n}$;
4. estimate the Bernstein copula by smoothing the empirical copula $C_{n}$ using the *m*^th^-degree multivariate Bernstein polynomial $B_{C_{n}}^{m}$;
5. obtain a continuous estimate of the multivariate distribution function ${\hat{F}}_{m,n}$ given by Equation (4);
6. discretize ${\hat{F}}_{m,n}$ using Equation (3) to obtain an estimate of the discrete multivariate probability mass function.

## 4. Applications

To evaluate the robustness of the method, we simulated datasets from two bivariate discrete distributions generated using copulas (Examples 4.1 and 4.2). For each simulated example, we present the estimated probabilities for three cases:

1. 600 pairs of observations with no censored data;
2. censored data in only one marginal, with 1000 observations in one marginal and 200 in another; and
3. censored data in both variables, with 600 observations for each variable, 300 of which form complete pairs.

After these examples, we present and compare estimates to the observed data from the OCD study (Example 4.3).

For the examples described above, we present the estimates for the probability mass functions considering the proposed method and compare its performance with some existing solutions, similar to those briefly discussed in Section 2 and which are more detailed below:

- the empirical distribution presented in Equation (1), that is obtained using only the complete pairs and the resulting probability function, coincides with the relative frequencies of the points observed in the sample;
- the multivariate skew t approximation that is obtained through a discretization of a parametric multivariate continuous distribution, estimated by the maximum likelihood method using only the complete pairs;
- the discretization of the normal copula with the normal marginal approximation to the distribution function that is obtained by using all observations for marginal distribution estimation and using only the complete pairs for copula estimation. This method is quite similar to that described in (b) considering the normal multivariate distribution rather than the skew t distribution, but here, it is possible to estimate the marginal distributions using all available data, not just the complete pairs;
- the discretization of the empirical Bernstein polynomial approximation presented in Equation (2), replacing the function *h* by the empirical distribution $F^{\left(n\right)}$ obtained in (a) and using only the complete pairs; and
- our proposed solution described in the previous section, which is obtained by using the Bernstein polynomial to approximate the margins using all observations and the approximated copula using the complete pairs.

For all examples, we graphically illustrate the estimates of the probability mass distributions and evaluate several distances between the estimated and theoretical distributions. For this purpose, some notation must be introduced. Let $\theta =\left(\theta_{1},\ldots ,\theta_{k}\right)$ be the theoretical probabilities, and let $\hat{\theta }=\left(\hat{\theta_{1}},\ldots ,\hat{\theta_{k}}\right)$ be the estimated probabilities. We consider the following distances for comparison of the estimates:

- Aitchison’s distance:
  $$\Delta \left(\hat{\theta },\theta \right)=\sqrt{\sum_{i=1}^{k}{\left[\ln\left(\frac{{\hat{\theta }}_{i}}{\theta_{i}}\right)-\bar{L}\right]}^{2}},\mathrm{where}\bar{L}=\frac{1}{k}\sum_{i=1}^{k}\ln\left(\frac{{\hat{\theta }}_{i}}{\theta_{i}}\right)$$
- Euclidean distance:
  $$\delta \left(\hat{\theta },\theta \right)=\sqrt{\sum_{i=1}^{k}{\left[{\hat{\theta }}_{i}-\theta_{i}\right]}^{2}}$$
- Total variation distance:
  $$\tau \left(\hat{\theta },\theta \right)=\frac{1}{2}\sum_{i=1}^{k}\left|{\hat{\theta }}_{i}-\theta_{i}\right|$$
- Kullback–Leibler symmetrized divergence:
  $$D\left(\hat{\theta },\theta \right)=\frac{1}{2}\left[\sum_{i=1}^{k}\theta_{i}\ln\left(\frac{\theta_{i}}{{\hat{\theta }}_{i}}\right)+\sum_{i=1}^{k}{\hat{\theta }}_{i}\ln\left(\frac{{\hat{\theta }}_{i}}{\theta_{i}}\right)\right]$$

Aitchison [39,40] and Pawlowsky [41] have presented many arguments for using Aitchison’s distance for compositional vectors, that is when the sum of the vector’s components is constant (in our case, the sum of the probabilities is equal to one). Moreover, the orderings implied by these distances agree in most cases.

At the end of this section, we present the estimates for the distribution of the real data described in the Introduction. In this case, we do not know the theoretical distribution; we present only the estimates and the distances calculated from the empirical distribution.

### 4.1. Simulated Elliptically-Shaped Distribution

In this section, we simulate data from an elliptically-shaped distribution with marginals $X_{1}∼\text{beta-binomial}\left(N_{x}=20,\alpha =5,\beta =5\right)$ and $Y_{1}∼\mathrm{binomial}\left(N_{y}=20,\pi =0.5\right)$ and a normal copula with parameter ρ=0.7.

**Figure 2:**
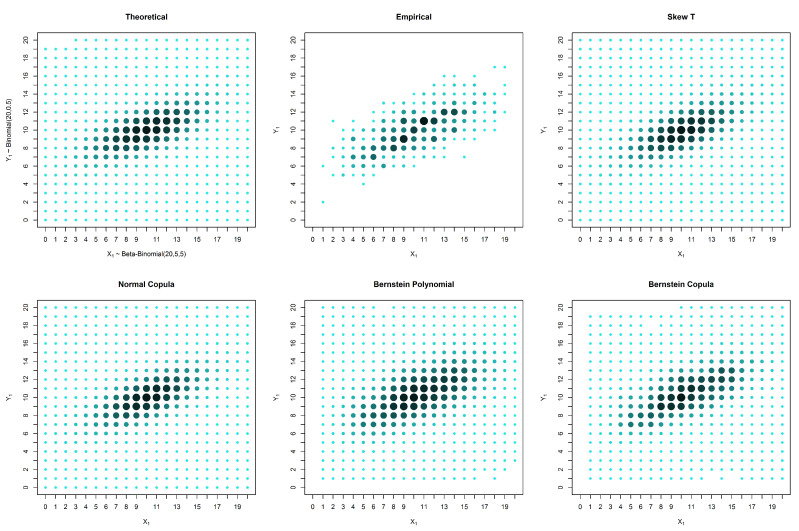
Estimates and theoretical probabilities for 600 complete pairs of observations, simulated from an elliptically-shaped distribution.

**Figure 3:**
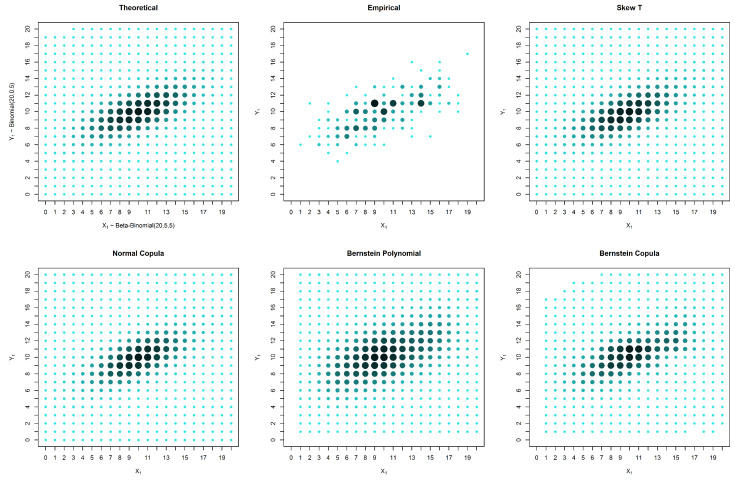
Estimates and theoretical probabilities for the case of censored data in only one marginal, with 1000 observations in one marginal and 200 in the other, simulated from an elliptically-shaped distribution.

**Figure 4:**
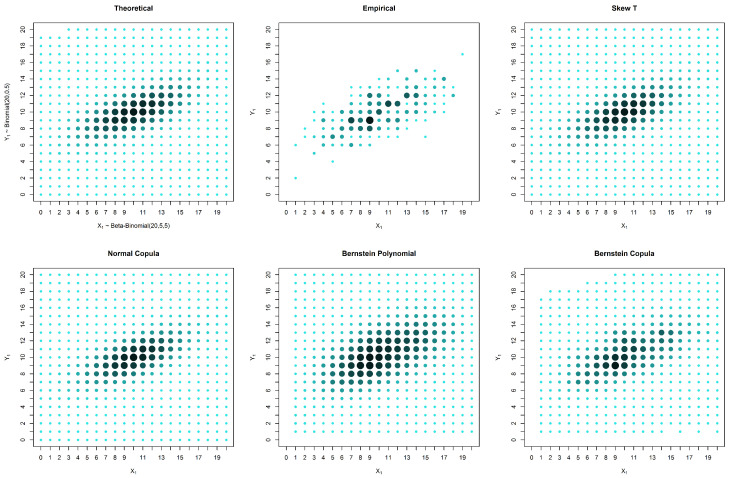
Estimates and theoretical probabilities for the case of censored data in both variables, with 600 observations for each variable, of which 300 form complete pairs, simulated from an elliptically-shaped distribution.

We can see from Figure 2, Figure 3 and Figure 4 and from Table 1, Table 2 and Table 3 that in these examples, the solutions based on elliptical distributions, namely the skew t and normal distributions, yield better estimates. This superior estimation occurs because the theoretical probability mass function is elliptical in shape. However, in practical situations, we have no knowledge of the real shape of the distribution. In such a case, the empirical distribution may be a good basis for evaluating the estimates, despite the existence of many unobserved points that are estimated as zero. When the estimates are compared with the empirical distribution, our proposed solution appears to produce good results, particularly in the presence of censored data.

### 4.2. Simulated Asymmetrical Distribution

In this section, we present the simulated data for an asymmetrical distribution with margins $X_{2}∼\mathrm{beta-binomial}\left(N_{x}=20,\alpha =0.85,\beta =1.1\right)$ and $Y_{2}∼\mathrm{binomial}\left(N_{y}=15,\pi =0.6\right)$ and a Gumbel copula with the parameter θ=0.7.

**Figure 5:**
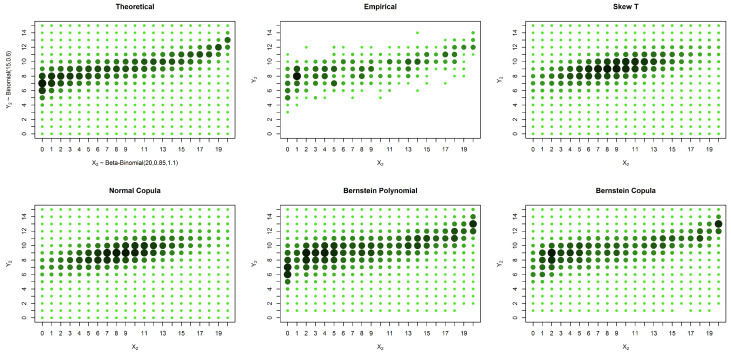
Estimates and theoretical probabilities for 600 complete pairs of observations, simulated from an asymmetrical distribution.

**Figure 6:**
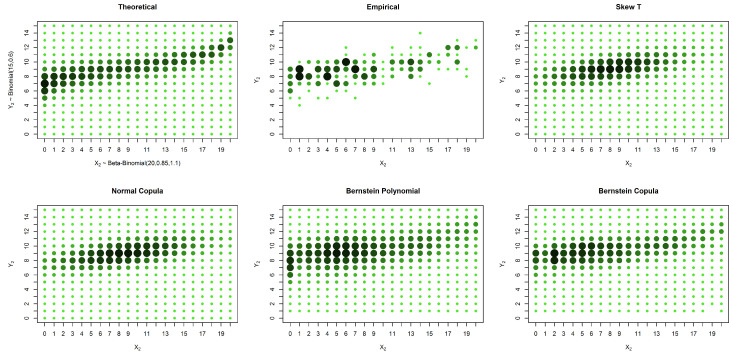
Estimates and theoretical probabilities for the case of censored data in only one marginal, with 1000 observations in one marginal and 200 in the other, simulated from an asymmetrical distribution.

**Figure 7:**
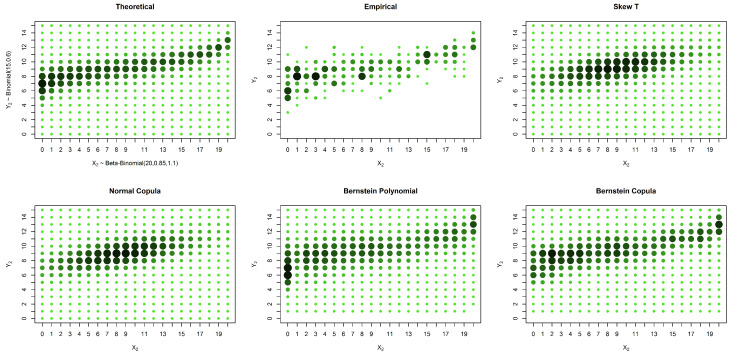
Estimates and theoretical probabilities for the case of censored data in both variables, with 600 observations for each variable, of which 300 form complete pairs, simulated from an asymmetrical distribution.

In the case of an asymmetrical distribution, our proposed solution yields a better estimation in all three considered cases: the case with no censored data, the case with missing data in one variable and the case with missing data in both variables. The superior performance of our approach can be observed both graphically (Figure 5, Figure 6 and Figure 7) and from the calculated distances (Table 4, Table 5 and Table 6). It is possible to graphically observe that the probabilities of both the smaller and larger values of $X_{2}$ are well estimated by our method.

**Figure 8:**
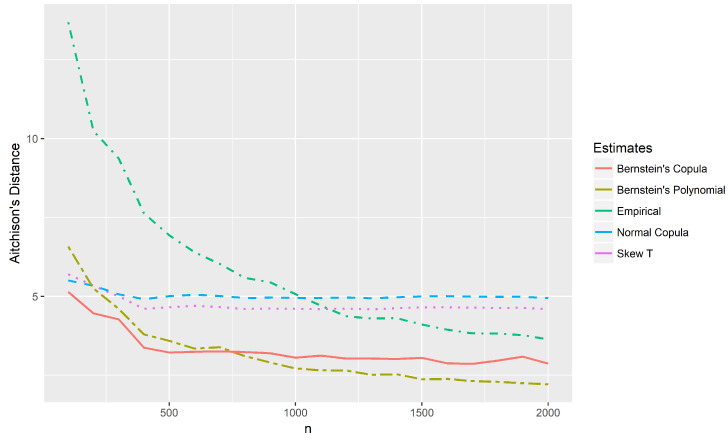
Distances between the estimates and theoretical probabilities for the case of censored data in only one marginal, with *n* observations in one marginal and 80% of censored data in the other one.

The new method performed better than the other presented solutions in the case of asymmetric models. It should be emphasized that the method was developed for cases with a large proportion of censored data or situations in which the number of points to be estimated is larger than the sample size. Figure 8 shows the Aitchison distances between the theoretical distribution and the estimates of the example of Section 4.2 considering censored data in only one marginal. Sample sizes n∈{1,…,2000} with the same proportion of censored data of the example were considered. In this case, the support of the distribution has 336 points. Note that for small samples (n<750), the proposed method presents the smallest distances. Although the method was developed for small samples, it appears that its estimates converge to the theoretical distribution as *n* increases. However, a detailed investigation on the asymptotic properties of the new method is needed and is the goal of a future work.

### 4.3. Real Data

In this section, we present the estimates for the real data described in the Introduction. The YBOCS is one of the most widely-used outcome measures in treatment studies of obsessive compulsive disorder (OCD). The total YBOCS scores comprise an integer number varying from 0–40 and intend to grade the severity of obsessive-compulsive symptoms. The total YBOCS score is the sum of two sub-scales, each ranging from 0–20, one of which measures the severity of compulsion and the other of obsession. The works in [8,9] propose that instead of the sum, it would be better to consider the maximum of these two sub-scales, called M-YBOCS. Thus, psychiatrists have been interested in better understanding the properties of this new scale, such as the probability distribution of M-YBOCS scores before and after patients have received some treatment for OCD.

As already mentioned in the Introduction, the dataset has 1001 observations of the scores at the initial time and only 213 at the final time. This happens because many patients drop out of treatment. The causes of drop out can be extremely different, such as a reduction in symptoms making the patient feel that he/she does not need treatment, or even worsen the symptoms, causing the patient to discredit the treatment. The small number of complete pairs in the database makes it difficult to estimate the joint distribution.

In real problems, there are few cases where the law of probability that generates the data is revealed. In such cases, a fairly common way to assess whether the proposed methods are adequate is to compare estimates with observed data. In predictive models, for example, it is common to verify some distance between predicted and observed values. In this way, we compare the distance between the estimates and the empirical probability function (which is the relative frequency of each observed point). The proposed solution yields smaller distances than do the existing approaches (Table 7).

**Figure 9:**
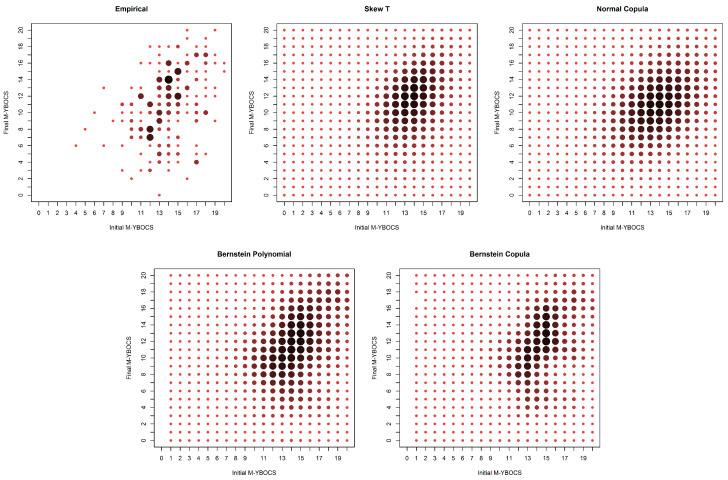
Estimates of probabilities for the real OCD data.

The estimation through the empirical distribution presents many zeros due to the small number of observations. The researchers believe that the proportion of unobserved points would decrease if the sample had fewer dropouts. In addition, they believe that common assumptions of normality or even symmetry assumptions make no sense in this case. The proposed method assigns positive probability to non-observed cells and captures the asymmetric nature of the data, which can be observed graphically in Figure 9.

## 5. Conclusions

In this work, a new approach to the problem of estimating discrete bivariate distributions is presented. The procedure, which essentially consists of estimating both the marginals and the copula using Bernstein polynomials, aims at addressing three important issues: the handling of discrete bivariate data in the presence of marginal missing values (using all available information, including incomplete pairs of observations); the possibility of obtaining positive estimates for non-observed cells, thus yielding “smoother” estimated discrete distributions; and the consideration of a large variety of dependence structures between the relevant random variables. The new approach is suitable for these cases owing to its fairly unrestrictive, nonparametric nature. The use of Bernstein polynomials shows better results of the empirical distribution to estimate the marginal distribution. It is important to note that the empirical Bernstein copula produces a copula only asymptotically [35], and other methods could be used to estimate Bernstein’s copula instead, as the one described in [42]. Anyway, the proposed method showed reasonable estimates for the studied probability mass functions. The new method can be applied also to *p*-dimensional random variables, p>2: both the mathematical development and the computational implementation are similar to the case p=2.

The new method was applied to several examples of simulated data, and according to a few typical measures of distance (between the estimated and theoretical distributions), it performed better than some of the existing solutions in cases of asymmetrical models, particularly in the presence of censored data and for cases where the number of points to be estimated is larger than the sample size. Although the method was developed for small samples, it appears that the proposed estimates converge to the theoretical distribution, but more detailed studies are still needed.

The new method was also applied to data sampled from adults diagnosed with primary obsessive-compulsive disorder. The estimate obtained by the method was appreciated by researchers in psychiatry.

While the new method has practical advantages over the presented existing alternatives, some aspects were not addressed here, namely: it will yet be necessary to further develop the new procedure in several aspects that were not addressed here: (i) the study of asymptotic properties for large sample size *n* and/or for higher polynomial degree *m*; (ii) a formal justification for the new procedure under a decision-theoretical approach; (iii) the development of a more rational approach to the selection of $m,m_{1},\ldots ,m_{p}$ (which could depend on *n*) using the approach suggested in (ii); and (iv) the incorporation of prior knowledge, perhaps as in Petrone [43,44] and Petrone and Wasserman [45], although these authors approached the problem from a univariate Bayesian perspective. These topics will be the focus of future articles.

## Figures and Tables

**Table 1:** Distances between the estimates and theoretical probabilities for 600 complete pairs of observations. The bold values highlight the smaller distances.

Example 4.1.1	Aitchison	Euclidean	Total Variation	Kullback–Leibler
Empirical	4.98521	0.02116	0.09988	0.04154
Skew T	1.44499	0.00629	0.02915	0.00345
Normal Copula	**1.28402**	**0.00476**	**0.02418**	**0.00236**
Bernstein Polynomial	3.45943	0.01388	0.07159	0.01870
Bernstein Copula	3.23712	0.01217	0.06360	0.01578

**Table 2:** Distances between the estimates and theoretical probabilities for the case of censored data in only one marginal, with 1000 observations in one marginal and 200 in the other. The bold values highlight the smaller distances.

Example 4.1.2	Aitchison	Euclidean	Total Variation	Kullback–Leibler
Empirical	8.97909	0.03454	0.17083	0.12490
Skew T	1.28441	**0.00493**	**0.02291**	**0.00239**
Normal Copula	**1.28040**	0.00554	0.02738	0.00284
Bernstein Polynomial	4.84901	0.02049	0.11110	0.03982
Bernstein Copula	3.28689	0.01171	0.06340	0.01530

**Table 3:** Distances between the estimates and theoretical probabilities for the case of censored data in both variables, with 600 observations for each variable, of which 300 form complete pairs. The bold values highlight the smaller distances.

Example 4.1.3	Aitchison	Euclidean	Total Variation	Kullback–Leibler
Empirical	7.32955	0.03035	0.13826	0.09162
Skew T	1.12383	0.00419	0.02221	0.00185
Normal Copula	**1.06365**	**0.00375**	**0.01891**	**0.00146**
Bernstein Polynomial	4.54073	0.01934	0.10051	0.03531
Bernstein Copula	3.54526	0.01377	0.06743	0.01963

**Table 4:** Distances between the estimates and theoretical probabilities for 600 complete pairs of observations. The bold values highlight the smaller distances.

Example 4.2.1	Aitchison	Euclidean	Total Variation	Kullback–Leibler
Empirical	6.17032	0.02767	0.12761	0.06377
Skew T	5.47625	0.03287	0.14429	0.07724
Normal Copula	5.76598	0.03293	0.14785	0.08020
Bernstein Polynomial	5.41325	**0.02436**	0.11969	0.05380
Bernstein Copula	**5.07634**	0.02519	**0.11842**	**0.05068**

**Table 5:** Distances between the estimates and theoretical probabilities for the case of censored data in only one marginal, with 1000 observations in one marginal and 200 in the other. The bold values highlight the smaller distances.

Example 4.2.2	Aitchison	Euclidean	Total Variation	Kullback–Leibler
Empirical	8.77534	0.03906	0.19104	0.14363
Skew T	5.23773	0.03130	0.13494	0.07356
Normal Copula	5.07437	0.02892	0.12727	0.06549
Bernstein Polynomial	5.65580	0.02626	0.13135	0.06562
Bernstein Copula	**4.86027**	**0.02558**	**0.11172**	**0.05379**

**Table 6:** Distances between the estimates and theoretical probabilities for the case of censored data in both variables, with 600 observations for each variable, of which 300 form complete pairs. The bold values highlight the smaller distances.

Example 4.2.3	Aitchison	Euclidean	Total Variation	Kullback–Leibler
Empirical	7.32005	0.03284	0.15576	0.09786
Skew T	4.79233	0.02760	0.12321	0.05917
Normal Copula	5.06253	0.02819	0.12855	0.06325
Bernstein Polynomial	5.09522	0.02253	0.11486	0.05028
Bernstein Copula	**4.35547**	**0.01957**	**0.09863**	**0.03595**

**Table 7:** Distances between the estimates and empirical probabilities for the real data.

Example 4.3	Aitchison	Euclidean	Total Variation	Kullback–Leibler
Skew T	11.56219	0.03941	0.22684	0.19899
Normal Copula	12.07092	0.04143	0.24701	0.21760
Bernstein	11.35361	0.03933	0.22910	0.19125
Bernstein Copula	**10.81475**	**0.03703**	**0.21020**	**0.17184**

## References

[1] Dos Anjos U.U., Ferreira F.H., Kolev N.V., Mendes B.M.V. (2004). Modeling Dependences via Copulas.

[2] Joe H. (1997). Multivariate Models and Dependence Concepts.

[3] Joe H. (2014). Dependence Modeling with Copulas.

[4] Nelsen R.B. (2006). An Introduction to Copulas.

[5] American Psychiatric Association (1994). Diagnostic and Statistical Manual of Mental Disorders (DSM-IV).

[6] Goodman W.K., Price L.H., Rasmussen S.A., Mazure C., Fleischmann R.L., Hill C.L., Heninger G.R., Charney D.S. (1989). The Yale-Brown Obsessive-Compulsive Scale: I. Development, use, and reliability. Arch. Gen. Psychiatry.

[7] Goodman W.K., Price L.H., Rasmussen S.A., Mazure C., Delgado P., Heninger G.R., Charney D.S. (1989). The Yale-Brown Obsessive-Compulsive Scale: II. Validity. Arch. Gen. Psychiatry.

[8] Pereira C.A.B., Silva C.B., Diniz J.B., Miguel E.C., Gentil V., Gattaz W.F. (2011). Estatística em Psiquiatria. Clínica Psiquiátrica.

[9] Diniz J.B., Fossaluza V., Belotto-Silva C., Shavitt R.G., Pereira C.A.B. (2011). The use of Yale-Brown Obsessive-Compulsive Scale: New views of an old measure. Eur. Neuropsychopharmacol..

[10] Johnson R.A., Wichern D.W. (2002). Applied Multivariate Statistical Analysis.

[11] Mardia K.V., Kent J.T., Bibby J.M. (1980). Multivariate Analysis.

[12] Branco M.D., Dey D.K. (2001). A General Class of Multivariate Skew-Elliptical Distributions. J. Multivar. Anal..

[13] Genton M.G., Loperfido N.M.R. (2005). Generalized skew-elliptical distributions and their quadratic forms. Ann. Inst. Stat. Math..

[14] Sklar A. (1959). Fonctions de répartition à n dimensions et leurs marges. Publ. Inst. Statistique Univ. Paris.

[15] Trivedi P.K., Zimmer D.M. (2007). Copula Modeling: An Introduction for Practitioners.

[16] Durrleman V., Nikeghbali A., Roncalli T. (2000). Which Copula Is the Right One.

[17] Fermanian J.D. (2005). Goodness-of-fit tests for copulas. J. Multivar. Anal..

[18] Genest C., Quessy J.F., Rémillard B. (2006). Goodness-of-fit procedures for copula models based on the probability integral transformation. Scand. J. Stat..

[19] Genest C., Rémillard B., Beaudoin D. (2009). Goodness-of-fit tests for copulas: A review and a power study. Insur. Math. Econ..

[20] Rakonczai P., Zempléni A., Skiadas C.H. (2007). Copulas and goodness of fit tests. Recent Advances in Stochastic Modeling and Data Analysis.

[21] Berg D. (2009). Copula goodness-of-fit testing: An overview and power comparison. Eur. J. Finance.

[22] DeVore R.A., Lorentz G.G. (1993). Constructive Approximation.

[23] Lorentz G.G. (1986). Bernstein Polynomials.

[24] Babu G.J., Canty A.J., Chaubey Y.P. (2002). Application of Bernstein polynomials for smooth estimation of a distribution and density function. J. Stat. Plan. Inference.

[25] Babu G.J., Chaubey Y.P. (2006). Smooth estimation of a distribution and density function on a hypercube using Bernstein polynomials for dependent random vectors. Stat. Probab. Lett..

[26] Heitzinger C., Hössinger A., Selberherr S. (2003). On smoothing three-dimensional Monte Carlo ion implantation simulation results. IEEE Trans. Comput. Aided Des. Integr. Circuits Syst..

[27] Heitzinger C., Hössinger A., Selberherr S. (2004). An algorithm for smoothing three-dimensional Monte Carlo ion implantation simulation results. Math. Comput. Simul..

[28] Li X., Mikusiński P., Sherwood H., Taylor M. (1997). Distributions with Given Marginals and Moment Problems.

[29] Li X., Mikusiński P., Taylor M.D. (1998). Strong approximations of copulas. J. Math. Anal. Appl..

[30] Kulpa T. (1999). On approximations of copulas. Int. J. Math. Math. Sci..

[31] Sancetta A., Satchell S.E. (2001). Bernstein Approximations to the Copula Function and Portfolio Optimization. https://ideas.repec.org/p/cam/camdae/0105.html.

[32] Taylor M.D. (2009). Bernstein polynomials and *n*-copulas. arXiv.

[33] Durrleman V., Nikeghbali A., Roncalli T. (2000). Copulas Approximation and New Families.

[34] Sancetta A. (2004). Nonparametric Estimation of Multivariate Distributions With Given Marginals. https://www.repository.cam.ac.uk/handle/1810/352.

[35] Sancetta A., Satchell S.E. (2004). The Bernstein copula and its applications to modeling and approximations of multivariate distributions. Econom. Theory.

[36] Sancetta A. (2007). Nonparametric estimation of distributions with given marginals via Bernstein–Kantorovich polynomials: *L*_1_ and pointwise convergence theory. J. Multivar. Anal..

[37] Bouezmarni T., Rombouts J.V.K., Taamouti A. (2010). Asymptotic properties of the Bernstein density copula estimator for *α*-mixing data. J. Multivar. Anal..

[38] Van der Vaart A.W., Wellner J.A. (1996). Weak cOnvergence and Empirical Processes.

[39] Aitchison J. (2003). The Statistical Analysis of Compositional Data.

[40] Aitchison J. (2008). The Single Principle of Compositional Data Analysis, Continuing Fallacies, Confusions and Misunderstandings and Some Suggested Remedies.

[41] Pawlowsky-Glahn V., Egozcue J.J., Tolosana-Delgado R. (2007). Lecture Notes on Compositional Data Analysis.

[42] Dou X., Kuriki S., Lin G.D., Richards D. (2016). EM algorithms for estimating the Bernstein copula. Comput. Stat. Data Anal..

[43] Petrone S. (1999). Random Bernstein polynomials. Scand. J. Stat..

[44] Petrone S. (1999). Bayesian density estimation using Bernstein polynomials. Can. J. Stat..

[45] Petrone S., Wasserman L. (2002). Consistency of Bernstein polynomial posteriors. J. R. Stat. Soc..

